# Towards Modified Entropy Mutual Information Feature Selection to Forecast Medium-Term Load Using a Deep Learning Model in Smart Homes

**DOI:** 10.3390/e22010068

**Published:** 2020-01-04

**Authors:** Omaji Samuel, Fahad A. Alzahrani, Raja Jalees Ul Hussen Khan, Hassan Farooq, Muhammad Shafiq, Muhammad Khalil Afzal, Nadeem Javaid

**Affiliations:** 1 Department of Computer Science, COMSATS University Islamabad, Islamabad 44000, Pakistan; omajiman1@gmail.com (O.S.); jalees106@gmail.com (R.J.U.H.K.);; 2 Computer Engineering Department, Umm AlQura University, Mecca 24381, Saudi Arabia; fayzahrani@uqu.edu.sa; 3 Department of Information and Communication Engineering, Yeungnam University, Gyeongsan 38541, Korea; 4 Department of Computer Science, COMSATS University Islamabad, Wah Cantonment 47040, Pakistan; khalilafzal@ciitwah.edu.pk

**Keywords:** big data analytics, conditional restricted Boltzmann machine, clustering analysis, dynamic behavior, jaya algorithm, medium-term load forecasting

## Abstract

Over the last decades, load forecasting is used by power companies to balance energy demand and supply. Among the several load forecasting methods, medium-term load forecasting is necessary for grid’s maintenance planning, settings of electricity prices, and harmonizing energy sharing arrangement. The forecasting of the month ahead electrical loads provides the information required for the interchange of energy among power companies. For accurate load forecasting, this paper proposes a model for medium-term load forecasting that uses hourly electrical load and temperature data to predict month ahead hourly electrical loads. For data preprocessing, modified entropy mutual information-based feature selection is used. It eliminates the redundancy and irrelevancy of features from the data. We employ the conditional restricted Boltzmann machine (CRBM) for the load forecasting. A meta-heuristic optimization algorithm Jaya is used to improve the CRBM’s accuracy rate and convergence. In addition, the consumers’ dynamic consumption behaviors are also investigated using a discrete-time Markov chain and an adaptive k-means is used to group their behaviors into clusters. We evaluated the proposed model using GEFCom2012 US utility dataset. Simulation results confirm that the proposed model achieves better accuracy, fast convergence, and low execution time as compared to other existing models in the literature.

## 1. Introduction

In the energy sector, load forecasting assists the utility to estimate the energy needed to balance energy supply and demand. In addition, load forecasting provides information that is used for easy energy interchange with other utilities. Long-term load forecasting (LTLF) of more than a year ahead is required to determine the grid’s regulatory policies and prices as well as the planning and construction of new electricity generation capacity. On the contrary, short-term load forecasting (STLF) of a few hours to couple of weeks ahead is required for economic planning of electricity generation capacity, security analysis, fuel purchases and short-term maintenance of grid. Very short-term load forecasting (VSTLF) of a few minutes to couple of hours ahead is required for real-time evaluation of security and system control. Medium-term load forecasting (MTLF) of few weeks to couple of months ahead is required for grid’s maintenance planning, settings of electricity prices and harmonization of the energy sharing arrangement. Researchers are actively working in LTLF and STLF; however, MTLF is not well explored. In this paper, we propose a MTLF method especially for month ahead load forecasting. Accurate electricity load forecasting is challenging because of the data collection and presentation. In addition, other challenges of load forecasting include: variations in electricity usage, unstable operating hours, seasonal trends, irregular weather conditions, and population growth.

We assume a utility company with a large dataset of electrical energy profiles, collected from the smart meters of several thousand residential homes. The dataset is non-uniform due to the nature of energy consumption patterns, appliances usage, smart meters collection methods, and uncertainty in pricing schemes. Analyzing the dataset requires suitable feature selection (FS) methods and load forecasting models for accurate forecasting. Due to the complexity of forecasting models, uncertainty and selection of FS methods, the performance of MTLF remains a key issue in decision making and economic development. In the literature, MTLF has been used  [1,2,3,4,5,6,7] for electricity load and price forecasting. However, FS methods have not gained enough attention in the area of MTLF; thus, this paper proposes a modified entropy mutual information (MI) FS method for MTLF. Note that FS is a method used in selecting the important feature variables that are necessary and efficient to train a forecasting model. Other applications of FS methods in STLF and VSTLF are given in [8,9,10,11,12,13,14,15,16,17,18]. Efficient FS methods enhance forecasting accuracy and make the forecasting model faster to train with less complexity. Thus, FS methods become one of the major factors for load forecasting.

Analyzing the behaviors of consumers is necessary to understand the benefit of balancing electricity demand and supply. Due to different household appliances and consumers’ load consumption preferences, consumers have different behaviors [19]. Neglecting the differences between consumers’ behaviors will undermine the improvement of the load forecasting accuracy. In addition, load profiles of different consumers during the same period may differ because each consumer has different household appliances and different time to use these appliances. Therefore, consumers with the same behaviors are clustered in the same group by extensively mining their behaviors, while consumers’ behaviors from different clusters are not the same [19]. All abbreviations used throughout this work are presented at the end of the paper.

This paper is the extension of our previous work [20]. The contributions of our paper are as follows.

1. We propose an entropy MI-based FS method that can handle both linear and nonlinear electricity load data; however, we improve the work of [21] to remove irrelevancy and redundancy of features. We also demonstrate how a modified entropy MI is applied to work systematically on load time series.
2. We propose auxiliary variables for the FS method based on four joint discrete random variables. Furthermore, the efficiency of the selected candidate features is examined based on ranking.
3. An accurate and robust MTLF (AR-MTLF) based on condition restricted Boltzmann machine (CRBM) is proposed to forecast month ahead hourly electrical loads. We refer to “robust” in our work to imply the efficiency of our proposed model in terms of execution time and the dynamic analysis of consumers’ behaviors. In addition, Jaya-based meta-heuristic optimization algorithm is used to improve the forecasting accuracy.
4. We analyze the consumers’ energy consumption behaviors by adopting a discrete time Markov chain (DTMC) that determines the state-dependent features. Furthermore, adaptive k-means [22] is used to classify electricity load into five groups, e.g., low, average, high, and extremely high consumption. In addition, it also derives the number of transitions and the obtained values serve as the quantization level of the load consumption.
5. The proposed model was implemented using the dataset of GEFCom2012 US utility [23]. In addition, we compared AR-MTLF model with accurate fast converging STLF (AFC-STLF) [21], artificial neural network (ANN), naive Bayes (NB), k-nearest neighbor (KNN), support vector regression (SVR), and ensemble models.

The paper is structured as follows. Section 2 provides the related work. Section 3 discusses the problem statement. In Section 4, the system model, MI-based FS, forecasting technique, and the dynamic consumers’ consumption behaviors are presented. Section 5 provides the simulation results and discussion. Finally, conclusion and future work are presented in Section 6.

## 2. Related Work

Today, MTLF is a method used in the smart grid for electricity price and load forecasting, although it has not gained full explorations. Contrarily, STLF is widely studied in [8,9,10,11,12,13,14,15,16,24], which imperatively introduce competitiveness in the electricity markets. Several methods of load forecasting begin from the conventional time series analysis to computational intelligence such as machine learning. The former method is linear based and the latter method is nonlinear based. Note that the conventional methods perform parameters estimation. In this section, the review of load forecasting techniques is presented; thereafter, MI-based FS methods followed by electricity users’ consumption behaviors are discussed.

The authors of [25] used regional electrical loads and weather parameters based on seasonally and weekly trends for short-term electrical load forecasting using regression tools (KNN regressor, linear regressor, and random forest). The authors combined Pearson’s method and visual inspection to analyze the relationship between the pre-processed data, test sets and simulation results. Mean absolute percentage error is used to examine the accuracy of the regressor models. Residual components of loads are generated from the total households’ load consumption, which are divided into predictable and non-predictable loads. The predictable loads have periodic behaviors, which enhance the accuracy of load forecasting. The authors of [26] proposed an adaptive circular conditional expectation model for operational scheduling of periodic loads and adaptive linear model for the short-term forecasting of residual loads. The forecasting accuracy of the proposed forecasting model was measured using a normalized mean absolute error. Note that the accurate results from load forecasting can improve the reliability of the power system. In retrospect, times series analysis and regression models are used for performing load forecasting; however, they are not efficient for the large households’ loads. The authors of [27] aggregated residential households’ loads for an efficient STLF using a deep neural network such as convolution neural network. The accuracy of the proposed forecasting model is examined using cumulative variation of root mean square error (RMSE) and mean absolute percentage error. Dong et al. [13] demonstrated STLF via a unit feature learning known as pyramid system and recurrent neural network (PRNN). The proposed system provides security and stability of the power grid. Forecasting is carried out on hourly historical load data using PRNN. The proposed system is compared to nine other methods of feature learning. In addition, two successful methods are used to evaluate the accuracy of forecasting intervals.

Price and load forecasting are imperative for electricity market competitiveness via optimal operation planning. However, many price and load forecasting methods have inefficient FS techniques to model interactive feature and nonlinear forecast processes. Abedinia et al. [24] proposed a new FS method by modeling the interaction between relevancy and redundancy with information theoretic criteria. A hybrid filter-wrapper approach is implemented to choose a minimum subset of maximum relevancy and minimum redundancy and maximum synergy of candidate features (MRMRMS) for short-term price forecasting (STPF) and STLF. Irena et al. [17] proposed a two-step method to obtain candidate features based on data categorization, correlation, and instance-based FS method. The proposed FS method is evaluated by auto-correlation, MI, RReliefF, and correlation, in addition, neural network (NN), linear regression (LR), and model tree rules (MTR) are also used. Auto-correlation-based FS methods and a robust NN forecasting algorithm are used to forecast loads, which show better performances as compared to the advanced exponential smoothing forecasting model. The authors of [28] used a quantile generalized additive model for non-operational and operational forecasting of load and price for STLF and MTLF. Their strategy forecasts the regressor variables, which are used in getting the operational forecasts. In addition, different temperature scenarios were considered for load forecasting. Furthermore, they performed electricity price forecasting by fitting a sparse linear regression to a large set of covariates.

The authors of [29] used CRBM and factor CRBM (FCRBM) for energy forecasting, which is classified by load profile based on measured data. Load forecasting is performed for 15-min, 1-h, and one-week time resolutions. The simulation results show that aggregated active power consumption gains the best forecasting results as compared to the load demand of intermittent appliances. However, the proposed network does not consider the weather temperature and other factors that may affect load forecasting. Quilumba et al. [30] resolved the effort involved in enhancing intra-day load forecasting using clustering method to detect the groups of consumers with similar load consumption from smart meters. K-means is used for clustering and NN is used for load forecasting. The authors also focused on the sub-hourly forecast with various time horizons up to one day ahead. Thus, the value of k in k-means must be known. However, it can create inaccurate clusters, if the number of k is chosen incorrectly. Similarly, the NN is prone to return solutions which are locally but not globally optimal. Singh et al. [31] proposed an intelligent model for data mining to forecast and analyze time series loads, which visually reveals several temporal consumption patterns. The operation of an appliance on hourly, weekly, monthly, and seasonally bases defines patterns that are used to examine consumers’ behavior. Unsupervised data clustering, frequent pattern analysis, and Bayesian network are trained to forecast the time series loads. In addition, the proposed model performs better than the support vector machine and multi-layer perceptron, respectively.

Smart meters are an important part of the power grid. The uses of smart meters’ data help to study and examine consumers’ behaviors. Yuancheng et al. [19] analyzed electricity behavior of consumers to achieve the accuracy of load forecasting. At first, individual daily loads of consumers are examined using various forecasting horizons, i.e., workdays, the day before the holidays, and holidays. Subsequently, electrical loads are grouped to classify consumers with the same behavior in a cluster. Furthermore, the electricity load forecasting of different groups is carried out using the online sequential extreme learning machine (ELM) (OS-ELM). This approach provides a summary of the entire system’s load as well as examines consumers’ electricity behaviors extensively. Therefore, it assures the accuracy of the load forecasting via clustering of consumers that uncovers the relationship between electricity behavior and cluster number. Although numerous works focus on STLF, in smart grid, VSTLF is imperatively used to solve, facilitate, and improve the quality of real-time electricity. Yu-Hsiang Hsiao [32] proposed a novel model for VSTLF that examines households’ data, based on daily scheduling patterns and context information. Distinctive behavioral patterns are used to examine the everyday electricity consumption and context features from different sources. In addition, it is used to control the anticipated behavioral patterns on a particular day. Thus, the volume of electricity consumption is modeled to predict individual’s behavioral patterns within a specific period of a day.

With the advent of the smart grid, many renewable energy resources such as solar and the wind are introduced into the power system. It creates intricate power system loads, which makes STLF analysis difficult. Pei et al. [33] proposed a STLF framework to address these limitations. The clustering analysis classifies the daily load patterns of different loads collected by smart meters. Afterwards, critical influential factors are determined by association analysis. Besides, the established classification criteria are applied via a decision tree. Finally, the best load forecasting models for an individual’s load patterns and the associated critical factors are selected. The selected models are used to examine the load forecasting by aggregating the different load forecasting results and line losses. Table 1 provides the summary of literature review in terms of the size of data, time resolutions, FS, techniques, and objectives.

## 3. Problem Statement

In the literature, several forecasting techniques mainly focus on conventional models such as fuzzy polynomial regression [8,9,11]. The conventional models are used to capture hidden information within the data. However, these models cannot solve complex nonlinear relationship between time series factors, i.e., daily time rhythm. Thus, it may cause substantial errors regarding load forecasting. In addition, these models do not pay sufficient attention to the time lags effect of external economic factors. Presently, machine learning techniques, such as ANNs, are used to forecast continuous time series and also provide adaptability [12,14,16,17]. However, the entire computational process is mostly a black box, which is not understandable compared to conventional methods.

Nevertheless, accuracy and convergence of machine learning techniques are not fully improved. For example, Liu et al. [34] proposed a hybrid ANN-based strategy to improve the forecasting accuracy. Despite this improvement, the entire strategy resulted in low convergence rate and high complexity. Similarly, the authors of [35] enhanced the convergence rate through ANN-based strategy, but achieved low forecasting accuracy. The authors of [36] further enhanced the work of [35] by incorporating an optimizer, which resulted in high execution time. In addition, Reference [21] improved the work in [35] by integrating a modified enhanced differential evolution algorithm (mEDE), modified MI-based FS and ANN. In MI-based FS process of [21], the downsized inputs do not further reduce the training time; here, information loss is observed. This is due to the unstable convergence of the mEDE and inefficiency of the model to train on massive amount of data.

In this paper, we improve the forecasting model of [21] by integrating CRBM. The preference of selecting CRBM over ANN is due to its ability to perform deep learning and it being a multi-layered neural network. In addition, CRBM uses the conditional hidden layer values as input to the next layer, whereas ANN is fine-tuned on whatever input it receives (i.e., label) and can be used as the traditional back propagation training method. CRBM is trained with a predetermined energy function, while ANN is trained by back propagation for achieving the least square objectives. In this paper, Jaya-based meta-heuristic optimization algorithm is used to minimize the forecasting error via iterative process. The choice of Jaya over the mEDE algorithm used in [21] is that mEDE requires parameters tuning, which may not guarantee the global optimum solution, whereas Jaya does not require algorithmic specific control parameters such as mutation and crossover rate. In this paper, we examine the dynamic behaviors of customers using adaptive k-means, which solves the problem of selecting k in k-means. In addition, a Markov chain (MC) is used to formulate the dynamic behavior of consumers, which indicates that the future energy consumption state correlates with the present states.

## 4. System Model

**Figure 1:**
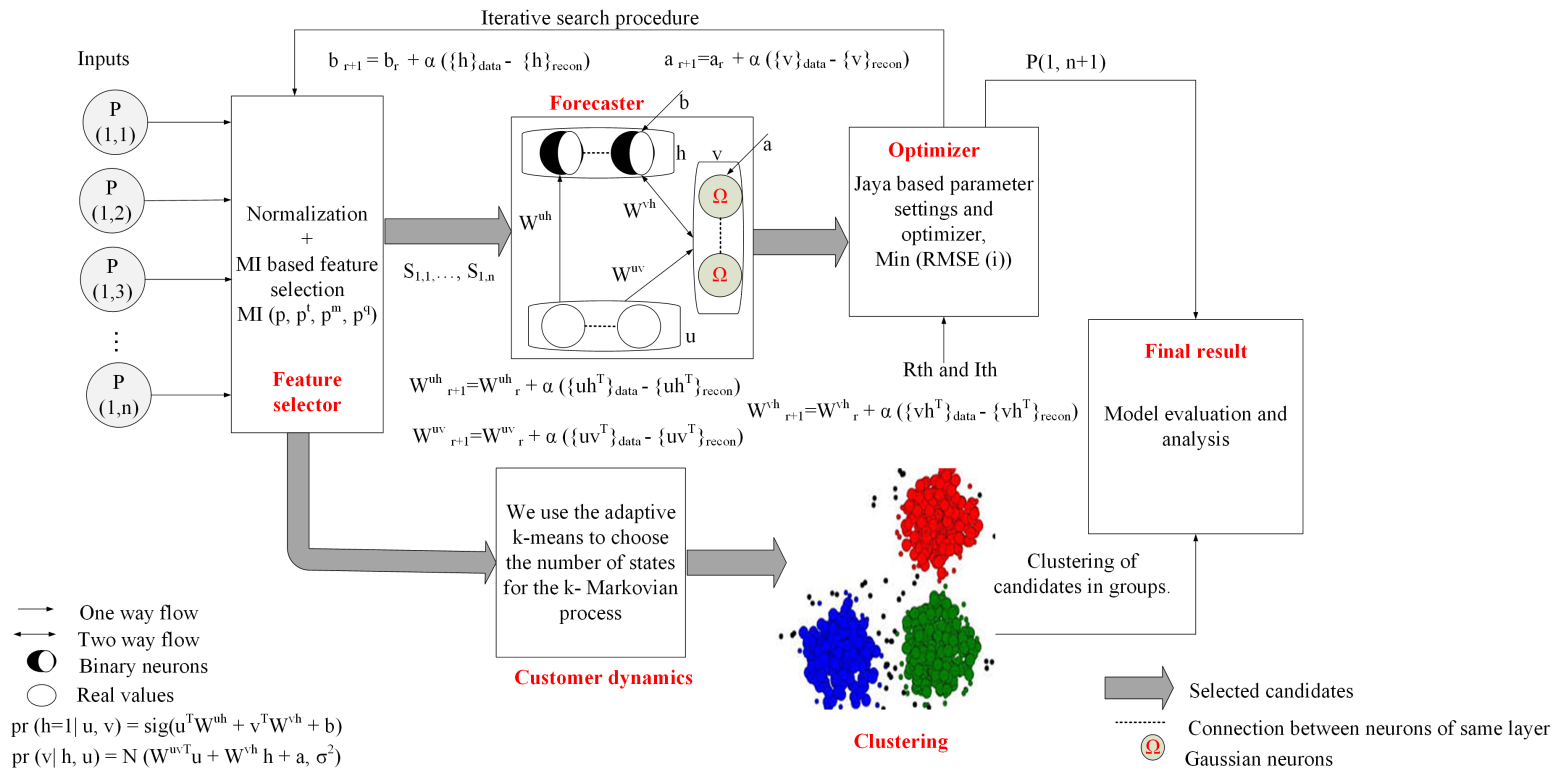
Proposed system model.

Figure 1 depicts our proposed system model, which consists of FS, forecaster, optimizer, and customer dynamics modules. At first, data of electrical loads are normalized. In the FS module, a modified entropy MI-based FS method is proposed to eliminate redundancy and irrelevancy from the data. It generates the candidate sets. In addition, candidates are sorted based on their ranking. Note that the candidates are designed based on target, average observed data, and moving average of data, which are partitioned into training, validation, and testing and used by the forecaster module for load forecasting. The forecasting error is minimized by the optimizer module through the iterative search process. DTMC is used to examine the consumers’ behaviors and the states indicate the patterns of load consumption as lowest, low, average, high, and extremely high. In Figure 1, *P* denotes the probability matrix of the input data; *u* denotes the input to the forecasting model; *W* denotes the random weight for the hidden, visible, and history layers, respectively; and forecasting model biases are denoted by “a” and “b” for visible and hidden layers. The number of iterations is denoted by *r*; α is the learning rate; $._{data}$ signifies the settings of forecasting model after it is fed with the training data; $._{recon}$ represents the settings of forecasting model after MC is performed; and *N* and sign denote the Gaussian and sigmoid functions, respectively.

### 4.1. Data Preparation and Preprocessing

**Figure 2:**
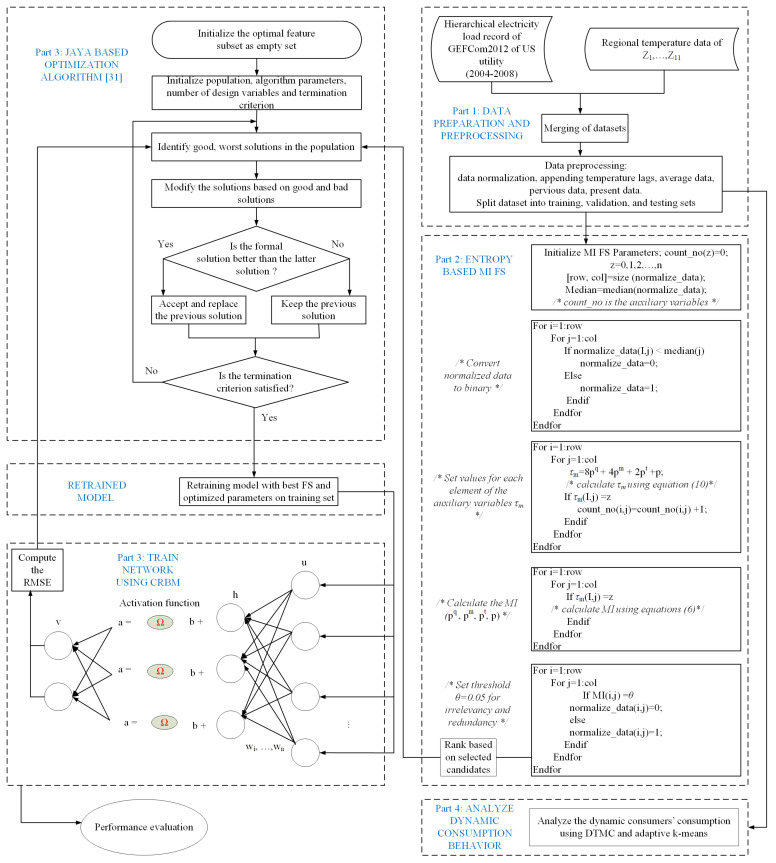
The system flowchart.

Figure 2 depicts the flowchart of our system model. Details of each step in the flowchart are presented in subsequent subsections of this paper. The processing step starts by combining the electrical load and the temperature data, which have an impact on the consumers’ electricity consumption behaviors. Based on this fact, the moving average of the load data $T_{h,d}$ of the *d*th day is calculated by Equation (1).

(1) $$\begin{array}{c} T_{h,d}=\frac{1}{24}\sum_{h=24d-23}^{24d}T_{d-h},\;d=1,2,\cdots ,z. \end{array}$$

The total time horizon is denoted by *z*. The preprocessing of data ensures zeroes and outliers are removed. In addition, the processed dataset is normalized to the range of [0, 1], while maintaining the temporal order. Three datasets are created from the normalized data for training, validation, and testing.

### 4.2. Modified MI Based FS

A survey by [37] provides the detailed discussion on the different types of FS techniques such as filter and wrapper methods. In the filter methods, ordering-based variable selection is used, which is constrained by variable ranking, e.g., correlation criteria and MI methods. In the wrapper methods, variable selections are performed by predictor, e.g., heuristic search algorithms and sequential selection algorithm. Other FS methods are semi-supervised learning, unsupervised learning, and ensemble FS. In this paper, a modified entropy-based MI FS method is proposed to eliminate redundancy and irrelevancy of features by choosing the best subsets for accurate load forecasting. In this way, the curse of dimensionality is prevented.

The proposed MI-based FS method consists of four joint discrete random variables defined in Equation (2).

(2) $$\begin{array}{c} MI\left(p,p^{t},p^{m},p^{q}\right)=\sum_{i}\sum_{j}\sum_{k}\sum_{l}pr\left(p,p^{t},p^{m},p^{q}\right)log_{2}\left(pr\left(\left(p,p^{t},p^{m},p^{q}\right)\right)\right), \end{array}$$

where the joint probability of the four discrete random variables are represented as $pr(p,p^{t},p^{m},p^{q})$, and pr(.) is a probability. Input discrete random variable is denoted as $p_{i}$, $p_{j}^{t}$ denotes the target value, $p_{k}^{m}$ represents the mean value, and $p_{l}^{q}$ denotes the moving average data. We formulate the proposed MI-based FS method as:

(3) $$\begin{array}{c} MI\left(p,p^{t},p^{m},p^{q}\right)=\sum_{i}\sum_{j}\sum_{k}\sum_{l}pr\left(p,p^{t},p^{m},p^{q}\right)log_{2}\left(\frac{pr(p,p^{t},p^{m},p^{q})}{pr\left(p\right)pr\left(p^{t}\right)pr\left(p^{m}\right)pr\left(p^{q}\right)}\right). \end{array}$$

If $MI(p,p^{t},p^{m},p^{q})=0$, it is independent. In addition, if $MI(p,p^{t},p^{m},p^{q})>0$, it is slightly related. Otherwise, if $MI(p,p^{t},p^{m},p^{q})<0$, it is not related. Ahmad et al. [21] added the target data as values of the previous day and average behavior to improve the forecasting results of their model. However, adding an average behavior is not sufficient. In this paper, temperature and moving average of the target data are included in the proposed forecasting model to achieve high forecasting accuracy. In Equation (3), the proposed MI is coded to binary values using Equation (4).

(4) $$\begin{array}{c} MI(p,p^{t},p^{m},p^{q})=\\ pr\left(p_{i}=0,p_{j}^{t}=0,p_{k}^{m}=0,p_{l}^{q}=0\right)\times log_{2}\left(\frac{pr\left(p_{i}=0\right),pr\left(p_{j}^{t}=0\right),pr\left(p_{k}^{m}=0\right),pr\left(p_{l}^{q}=0\right)}{pr\left(p_{i}=0\right)pr\left(p_{j}^{t}=0\right)pr\left(p_{k}^{m}=0\right)pr\left(p_{l}^{q}=0\right)}\right)\\ +\\ pr\left(p_{i}=0,p_{j}^{t}=0,p_{k}^{m}=0,p_{l}^{q}=1\right)\times log_{2}\left(\frac{pr\left(p_{i}=0\right),pr\left(p_{j}^{t}=0\right),pr\left(p_{k}^{m}=0\right),pr\left(p_{l}^{q}=1\right)}{pr\left(p_{i}=0\right)pr\left(p_{j}^{t}=0\right)pr\left(p_{k}^{m}=0\right)pr\left(p_{l}^{q}=1\right)}\right)\\ +\\ pr\left(p_{i}=0,p_{j}^{t}=0,p_{k}^{m}=1,p_{l}^{q}=0\right)\times log_{2}\left(\frac{pr\left(p_{i}=0\right),pr\left(p_{j}^{t}=0\right),pr\left(p_{k}^{m}=1\right),pr\left(p_{l}^{q}=0\right)}{pr\left(p_{i}=0\right)pr\left(p_{j}^{t}=0\right)pr\left(p_{k}^{m}=1\right)pr\left(p_{l}^{q}=0\right)}\right)\\ +\\ pr\left(p_{i}=0,p_{j}^{t}=0,p_{k}^{m}=1,p_{l}^{q}=1\right)\times log_{2}\left(\frac{pr\left(p_{i}=0\right),pr\left(p_{j}^{t}=0\right),pr\left(p_{k}^{m}=1\right),pr\left(p_{l}^{q}=1\right)}{pr\left(p_{i}=0\right)pr\left(p_{j}^{t}=0\right)pr\left(p_{k}^{m}=1\right)pr\left(p_{l}^{q}=1\right)}\right)\\ +\\ pr\left(p_{i}=0,p_{j}^{t}=1,p_{k}^{m}=0,p_{l}^{q}=0\right)\times log_{2}\left(\frac{pr\left(p_{i}=0\right),pr\left(p_{j}^{t}=1\right),pr\left(p_{k}^{m}=0\right),pr\left(p_{l}^{q}=0\right)}{pr\left(p_{i}=0\right)pr\left(p_{j}^{t}=1\right)pr\left(p_{k}^{m}=0\right)pr\left(p_{l}^{q}=0\right)}\right)\\ +\\ pr\left(p_{i}=0,p_{j}^{t}=1,p_{k}^{m}=0,p_{l}^{q}=1\right)\times log_{2}\left(\frac{pr\left(p_{i}=0\right),pr\left(p_{j}^{t}=1\right),pr\left(p_{k}^{m}=0\right),pr\left(p_{l}^{q}=1\right)}{pr\left(p_{i}=0\right)pr\left(p_{j}^{t}=1\right)pr\left(p_{k}^{m}=0\right)pr\left(p_{l}^{q}=1\right)}\right) \end{array}$$

(5) $$\begin{array}{c} +\\ pr\left(p_{i}=0,p_{j}^{t}=1,p_{k}^{m}=1,p_{l}^{q}=0\right)\times log_{2}\left(\frac{pr\left(p_{i}=0\right),pr\left(p_{j}^{t}=1\right),pr\left(p_{k}^{m}=1\right),pr\left(p_{l}^{q}=0\right)}{pr\left(p_{i}=0\right)pr\left(p_{j}^{t}=1\right)pr\left(p_{k}^{m}=1\right)pr\left(p_{l}^{q}=0\right)}\right)\\ +\\ pr\left(p_{i}=0,p_{j}^{t}=1,p_{k}^{m}=1,p_{l}^{q}=1\right)\times log_{2}\left(\frac{pr\left(p_{i}=0\right),pr\left(p_{j}^{t}=1\right),pr\left(p_{k}^{m}=1\right),pr\left(p_{l}^{q}=1\right)}{pr\left(p_{i}=0\right)pr\left(p_{j}^{t}=1\right)pr\left(p_{k}^{m}=1\right)pr\left(p_{l}^{q}=1\right)}\right)\\ +\\ pr\left(p_{i}=1,p_{j}^{t}=0,p_{k}^{m}=0,p_{l}^{q}=1\right)\times log_{2}\left(\frac{pr\left(p_{i}=1\right),pr\left(p_{j}^{t}=0\right),pr\left(p_{k}^{m}=0\right),pr\left(p_{l}^{q}=1\right)}{pr\left(p_{i}=1\right)pr\left(p_{j}^{t}=0\right)pr\left(p_{k}^{m}=0\right)pr\left(p_{l}^{q}=1\right)}\right)\\ +\\ pr\left(p_{i}=1,p_{j}^{t}=0,p_{k}^{m}=0,p_{l}^{q}=0\right)\times log_{2}\left(\frac{pr\left(p_{i}=1\right),pr\left(p_{j}^{t}=0\right),pr\left(p_{k}^{m}=0\right),pr\left(p_{l}^{q}=0\right)}{pr\left(p_{i}=1\right)pr\left(p_{j}^{t}=0\right)pr\left(p_{k}^{m}=0\right)pr\left(p_{l}^{q}=0\right)}\right)\\ +\\ pr\left(p_{i}=1,p_{j}^{t}=0,p_{k}^{m}=1,p_{l}^{q}=0\right)\times log_{2}\left(\frac{pr\left(p_{i}=1\right),pr\left(p_{j}^{t}=0\right),pr\left(p_{k}^{m}=1\right),pr\left(p_{l}^{q}=0\right)}{pr\left(p_{i}=1\right)pr\left(p_{j}^{t}=0\right)pr\left(p_{k}^{m}=1\right)pr\left(p_{l}^{q}=0\right)}\right)\\ +\\ pr\left(p_{i}=1,p_{j}^{t}=0,p_{k}^{m}=1,p_{l}^{q}=1\right)\times log_{2}\left(\frac{pr\left(p_{i}=1\right),pr\left(p_{j}^{t}=0\right),pr\left(p_{k}^{m}=1\right),pr\left(p_{l}^{q}=1\right)}{pr\left(p_{i}=1\right)pr\left(p_{j}^{t}=0\right)pr\left(p_{k}^{m}=1\right)pr\left(p_{l}^{q}=1\right)}\right)\\ +\\ pr\left(p_{i}=1,p_{j}^{t}=1,p_{k}^{m}=0,p_{l}^{q}=0\right)\times log_{2}\left(\frac{pr\left(p_{i}=1\right),pr\left(p_{j}^{t}=1\right),pr\left(p_{k}^{m}=0\right),pr\left(p_{l}^{q}=0\right)}{pr\left(p_{i}=1\right)pr\left(p_{j}^{t}=1\right)pr\left(p_{k}^{m}=0\right)pr\left(p_{l}^{q}=0\right)}\right)\\ +\\ pr\left(p_{i}=1,p_{j}^{t}=1,p_{k}^{m}=0,p_{l}^{q}=1\right)\times log_{2}\left(\frac{pr\left(p_{i}=1\right),pr\left(p_{j}^{t}=1\right),pr\left(p_{k}^{m}=0\right),pr\left(p_{l}^{q}=1\right)}{pr\left(p_{i}=1\right)pr\left(p_{j}^{t}=1\right)pr\left(p_{k}^{m}=0\right)pr\left(p_{l}^{q}=1\right)}\right)\\ +\\ pr\left(p_{i}=1,p_{j}^{t}=1,p_{k}^{m}=1,p_{l}^{q}=0\right)\times log_{2}\left(\frac{pr\left(p_{i}=1\right),pr\left(p_{j}^{t}=1\right),pr\left(p_{k}^{m}=1\right),pr\left(p_{l}^{q}=0\right)}{pr\left(p_{i}=1\right)pr\left(p_{j}^{t}=1\right)pr\left(p_{k}^{m}=1\right)pr\left(p_{l}^{q}=0\right)}\right)\\ +\\ pr\left(p_{i}=1,p_{j}^{t}=1,p_{k}^{m}=1,p_{l}^{q}=1\right)\times log_{2}\left(\frac{pr\left(p_{i}=1\right),pr\left(p_{j}^{t}=1\right),pr\left(p_{k}^{m}=1\right),pr\left(p_{l}^{q}=1\right)}{pr\left(p_{i}=1\right)pr\left(p_{j}^{t}=1\right)pr\left(p_{k}^{m}=1\right)pr\left(p_{l}^{q}=1\right)}\right). \end{array}$$

**Figure 3:**
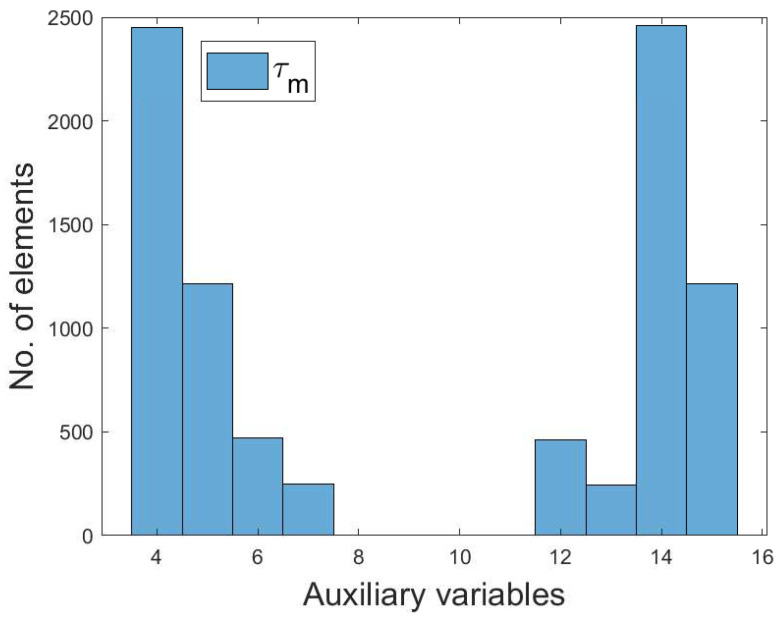
Simulation of auxiliary variables.

We introduce an auxiliary variable $\tau_{m}$ for the individual elements and the joint probability is given in Equation (6).

(6) $$\begin{array}{c} \tau_{m}=8p^{q}+4p^{m}+2p^{t}+p, \end{array}$$

where $\tau_{m}\in \left[0,1,\cdots ,15\right]$. $\tau_{0m}$ is the number of zeros, $\tau_{1m}$ is the number of ones, $\tau_{2m}$ is the number of twos, etc. Figure 3 reports the simulation results for the fifteen auxiliary variables. Note that the empty spaces of auxiliary variables 8, 9, 19, and 11 depict that there are no corresponding matching elements. The joint probability of the individual value of $\tau_{m}$ is calculated using Equation (7).

(7) $$\begin{array}{c} pr\left(p=0\right)=\frac{\tau_{0m}+\tau_{2m}+\tau_{4m}+\tau_{6m}+\tau_{8m}+\tau_{10m}+\tau_{12m}+\tau_{14m}}{L}\\ pr\left(p=1\right)=\frac{\tau_{1m}+\tau_{3m}+\tau_{5m}+\tau_{7m}+\tau_{9m}+\tau_{11m}+\tau_{13m}+\tau_{15m}}{L}\\ pr\left(p^{t}=0\right)=\frac{\tau_{0m}+\tau_{1m}+\tau_{2m}+\tau_{3m}+\tau_{8m}+\tau_{9m}+\tau_{10m}+\tau_{11m}}{L}\\ pr\left(p^{t}=1\right)=\frac{\tau_{4m}+\tau_{5m}+\tau_{6m}+\tau_{7m}+\tau_{12m}+\tau_{13m}+\tau_{14m}+\tau_{15m}}{L}\\ pr\left(p^{m}=0\right)=\frac{\tau_{0m}+\tau_{1m}+\tau_{4m}+\tau_{5m}+\tau_{8m}+\tau_{9m}+\tau_{12m}+\tau_{13m}}{L} \end{array}$$

(8) $$\begin{array}{c} pr\left(p^{m}=1\right)=\frac{\tau_{2m}+\tau_{3m}+\tau_{6m}+\tau_{7m}+\tau_{10m}+\tau_{11m}+\tau_{14m}+\tau_{15m}}{L}\\ pr\left(p^{q}=0\right)=\frac{\tau_{0m}+\tau_{1m}+\tau_{2m}+\tau_{3m}+\tau_{4m}+\tau_{5m}+\tau_{6m}+\tau_{7m}}{L}\\ pr\left(p^{q}=1\right)=\frac{\tau_{8m}+\tau_{9m}+\tau_{10m}+\tau_{11m}+\tau_{12m}+\tau_{13m}+\tau_{14m}+\tau_{15m}}{L}. \end{array}$$

In the proposed MI-based FS method in Equation (7), *L* denotes the length of input data. The candidates are sorted based on the values of MI. With the the sorted values, redundancy and irrelevancy of features are eliminated. Based on the proposed forecasting model, the selected candidates, $S_{1,1},\cdots ,S_{1,n}$, are coded to binary values using Equation (4).

### 4.3. Forecaster Module

The forecaster module depicted in Figure 1 shows the configuration of CRBM model, which is adopted from [29]. In our proposed model, CRBM performs three steps: cost function minimization, gradient update, and probability inference. Interested readers can find the details of these steps in the work by [29]. The aim of our proposed forecasting model is to forecast the energy consumption for a given time slot or series of time slots in the future. By obtaining the historical energy consumption data, the time slots between the two measurements should be the same (i.e., input and output). We consider the time slot *k* for the historical energy consumption as the current data. Here, the vector that denotes the historical energy consumption data is expressed as:

(9) $$P_{k}=\left\{p_{1},p_{2},\cdots ,p_{k-1}\right\},$$

where $P_{k}$ denotes the *k*th measurement. The forecasting model should be able to forecast the energy consumption for the next *h* time slot, which is expressed as:

(10) $$\bar{P_{k}}=\left\{\bar{p_{k}},\bar{p_{k+1}},\cdots ,\bar{p_{k+h-1}}\right\},$$

where $\bar{P_{k}}$ denotes the *k*th measurement for the forecasted energy consumption data. The input vector of our proposed AR-MTLF model is expressed as:

(11) $$I_{k}=\left\{O_{k},p_{k}^{q},p_{k}^{m},p_{k}^{t},p_{k},F_{k}\right\},$$

where $I_{k}$ is the *k*th input to the hidden layer of the forecaster module, $O_{k}$ is the *k*th output from the forecaster module, $p_{k}^{q}$ is the *k*th moving average value, $p_{k}^{m}$ is the *k*th mean value, $p_{k}^{t}$ is the *k*th target values, $p_{k}$ is the *k*th historical value, and $F_{k}$ is the *k*th flag that defines if the first forecasting time slot is on the weekend. Note that, if the historical data for previous four time slots are used as input to make the forecasting for the next time slot, the fifth time slot is used as the input to the hidden layer of the forecasting model.

To evaluate the accuracy of the proposed forecasting model, RMSE is used.

(12) $$\begin{array}{c} RMSE\left(j\right)=\sqrt{\frac{1}{N}\sum_{k=1}^{N}{\left(A_{k}-A_{k}^{{}^{\prime }}\right)}^{2}}, \end{array}$$

where $A_{k}$ represents the *k*th actual load and $A_{k}^{{}^{\prime }}$ denotes the *k*th predicted load. The value of *N* is used to denote the time trends such as hourly, daily, weekly, and monthly trends. Note that after, sequences of iterations, the final value of RMSE becomes the validation error.

### 4.4. Optimizer Module

In this paper, the forecasting model’s accuracy is improved using Jaya optimization algorithm, which is adopted from [38]. It has been used to solve the non-constrained and constrained optimization problems [39]. We define the objective function as:

(13) $$\begin{array}{c} \begin{array}{c} Minimize \end{array}\;\;RMSE\left(j\right)\;\forall \;j\in \left[1,2,\cdots ,N\right], \end{array}$$

where *N* is the total time horizon. Table 2 provides the simulation parameters of the optimizer. Note that this paper aims at achieving, firstly, fast convergence, i.e., the time spent by the system during simulation to execute the forecasting strategy and, secondly, acceptable minimum forecasting error (the system is able to forecast in the fastest manner).

### 4.5. Customers’ Dynamic Behavior

**Figure 4:**
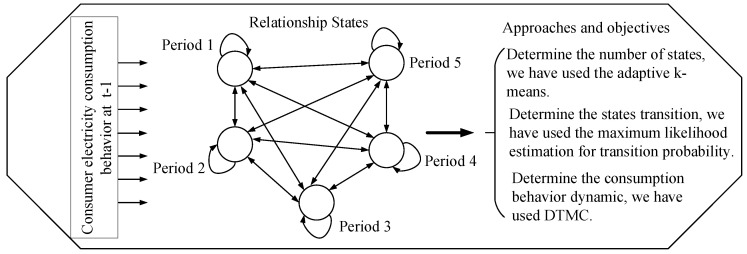
Approaches to consumers’ dynamic energy consumption.

In this section, we discuss the consumers’ dynamic energy consumption behaviors using the approach shown in Figure 4. The related work on load profiling mainly focuses on a single residential customer, which shows a weak regularity. It is important to note that the dynamic characteristics are best in combined consumers and can be illustrated using different consumers’ load profiles. However, due to the randomness of these different consumers’ load profiles, the real consumption behavior of consumers cannot be efficiently examined. To handle the problem, a DTMC is used to formulate the dynamic behavior of consumers by considering the state-dependent features. Thus, it indicates that the future load consumption behaviors of consumers will correlate with their present states.

The relations and transitions between consumption behaviors in adjacent periods are known as dynamics [40]. In this paper, recording the dynamics as a factor of grouping is required, which is useful in deriving vital information about the consumers’ consumption patterns within the shortest period of time. It also helps to establish the potential demand response target and reduces the dimensionality of the dataset.

**Figure 5:**
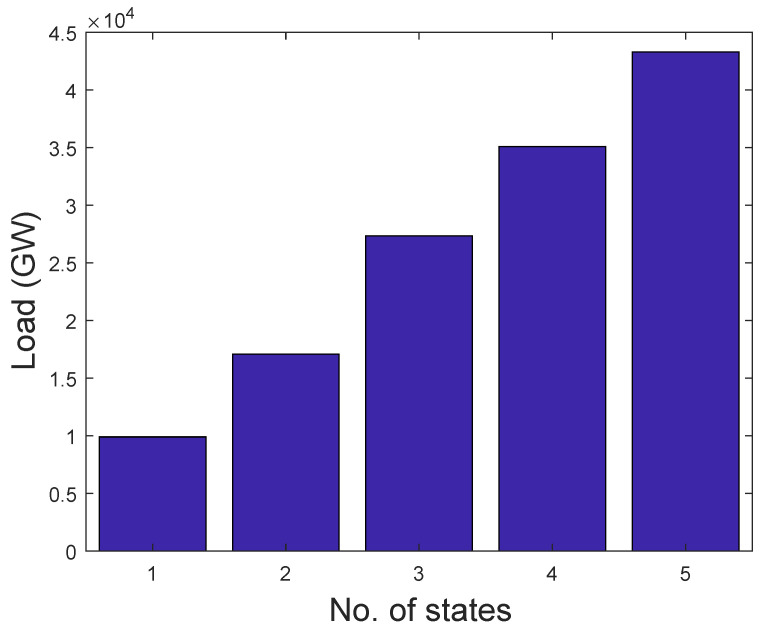
Load categorization.

Based on the load profile, this paper uses adaptive k-means from [41] to deduce the number of k-Markovian states. With respect to this, we classify consumers’ consumption into five states, namely lowest, low, average, high, and extremely high, which are denoted by 1–5 in Figure 5, respectively. To group the consumption into a well-defined classification, a k-centroid obtained from the adaptive k-means is used to ascertain the required k-states. The value of k-means serves as the quantization level of load profile and the quantize values are used to derive the number of transitions. Algorithm 1 illustrates the proposed adaptive k-means. To determine the state transition probability, we use the maximum likelihood estimation, which is given in Equation (14) [42].

(14) $$\begin{array}{c} pr_{x,i}=\frac{n_{x,i}}{\sum_{i=1}^{t}n_{x}}, \end{array}$$

where $n_{x,i}$ is the number of transitions from state *x* to state *i* and $\sum_{i=1}^{t}n_{x}$ is the total number of transitions from state *x*. In Table 3, a five-step transition probability is obtained by transitioning from state *x* to state *i* in a single step and, in addition, it is a transition matrix table with a square matrix. When $pr_{5,2}=0.0000$, it means that, if you start from the state *x* with row=5 to state *i* on column=2, the transition probability is zero. On the other hand, it means that, if you start from state *i* with column=2 to state *x* on row=5, then the transition probability is zero. Hence, whichever state the consumers start with, their transition probabilities are zero, which means that the consumers are absorbed in that state. MC will either stay at the current state or move to the adjacent state. The transition probability is used to examine the current behavior of the consumer for decision making.
**Algorithm 1** Adaptive k-means.1: **procedure**adaptive k-means(actual dataset)
2:    i←0;,j←0;▹ initialize iteration counter3:    data←double(actualdataset);
4:    index←data(:);▹ copy value as array5:    **while**
true
**do**
6:        $M_{1}\leftarrow mean\left(data\right)$▹ initialize the mean point7:        i←i+1;▹ increment counter for each iteration8:        **while**
true
**do**
9:           j←j+1;▹ increment counter for each iteration10:           $ds\leftarrow {\left(index-M_{1}\right)}^{2};$▹ find the distance between index and data11:           $ds_{1}\leftarrow \left(sum{\left(index-M_{1}\right)}^{2}/N\right);$▹ N is the number of dataset12:           $best\leftarrow ds-ds_{1};$▹ check whether it is selected accurately13:           $M_{1}^{new}\leftarrow mean\left(M_{1}\left(best\right)\right);$▹ update mean point14:           **if**
$M_{1}^{new}$
**then**
15:               **break;**
16:           **end if**
17:           **if**
$M_{1}==M_{1}^{new}\;|\;j>\beta$
**then**▹ β is distortion threshold18:               j←0;
19:               index(best)←[];▹ remove value that is already assigned to a cluster20:               $Center\left(i\right)\leftarrow M_{1}^{new}$▹ store center of cluster21:               **break;**
22:           **end if**
23:           $M_{1}\leftarrow M_{1}^{new}$▹ update mean point24:        **end while**
25:        **if**
index==0|i>β
**then**▹ check maximum number of cluster26:           i←0;
27:           **break;**
28:        **end if**
29:    **end while**
30:    Center←sort(Center);▹ sort center31:    $Center^{new}\leftarrow diff\left(Center\right);$▹ find the differences between two centers32:    $Center\left(Center^{new}<=intercluster\right)=\left[\;\right];$▹ ignore cluster center less than distance33:    distance←data−Center;▹ find distance between cluster and data34:    [,indx]←min(distance)▹ choose cluster index of minimum distance35:    **return**
indx,Center
36:
**end procedure**



The choice of selecting a better distance function is challenging and the attempt to develop a cluster analysis on the dataset may result in different distance function values. Choosing a wrong distance function may not capture the variability of the data correctly. For example, based on the experiment and survey conducted by the authors of [43,44,45], we conclude that distance values differ across different distance functions. Distance function calculation plays a vital role in the clustering algorithm, as the distances between two points depend on the properties of the data as well as the dimension of the dataset. In addition, when random initialization of centroid is used, different simulations of k-means will introduce a different sum of square error (SSE). It is shown from the experimental studies of [44,45] that the number of iterations in Euclidean distance function is more than the Manhattan distance function, which makes the k-means less computationally time complex. Besides, the city block distance function shows better performance on both datasets (iris and wine) in terms of computational time as compared to Euclidean and Manhattan distance functions. A well-known distance function is Euclidean distance that is used to analyze all continuous numerical variables that reflect absolute distances. However, it does not remove redundancy. Mahalanobis distance is also a popular distance function that is used when there are continuous numerical variables that reflect absolute distances as well as to remove redundancy. Besides, if we are concerned about making a distinction between variables, the family of Hellinger distance, species profile, and chord distance are appropriate distance functions. These distances are weighted by the overall quantity of each sample. Hence, smaller distances are obtained when the variables of each sample are similar while the absolute magnitude varies. In this paper, we use the squared Euclidean distance, where each centroid is the mean point in the cluster, as defined in Equation (15).

(15) $$\begin{array}{c} ds=\sum_{i=1}^{M}{\left(x-c\right)}^{2}, \end{array}$$

where *x* is the observation, *c* is the centroid, and the total number of observations is denoted by *M*. The importance of using the squared Euclidean distance is to avoid computing the square root that derives the squared distance between two data points. In addition, it saves computation costs. In the DTMC, we consider the random variables $Y_{1},Y_{2},Y_{3},\cdots$, with Markov property, which states that the probability of moving to the next state depends only on the present state and not the previous state [42]. We verify this fact in our simulation by assuming a static state of the MC, and then we record the transition of the state using Equation (16).

(16) $$\begin{array}{c} pr(Y_{n+1}=y\;|\;Y_{1}=y_{1},Y_{2}=y_{2},\cdots ,Y_{n}=y_{n})\\ =pr(Y_{n+1}=y|Y_{n}=y_{n}). \end{array}$$

If both conditions are well defined, i.e., $pr(Y_{n+1}=y|Y_{n}=y_{n})>0$, then the values of *Y* are the state space. MC is mostly explained by a directed graph, where the edge *n* is the probability of going from one state at time *n* to another state at time n+1. This can also be represented using a transition matrix from time *n* to n+1. MC can be assumed to be time-homogeneous, by which the matrices are independent of *n*. Time-homogeneous can be described as a state machine that assigns a probability of moving from a state to an adjacent one [42]. Thus, the probability can be studied as the statistical behavior of the machine’s state with the first element of the state space as input given below.

(17) $$\begin{array}{c} pr\left(Y_{n+1}=y_{n}\;|\;Y_{1}=x\right)=pr\left(Y_{n}=y\;|\;Y_{n-1}=x\right)\;\forall \;n. \end{array}$$

The probability of transition does not depend on *n*. MC can also be described as MC with memory, in which the future state depends on the past state. Certain properties of MC [42] are relevant to discuss in this paper as follows.

1. Irreducible: MC is irreducible if all states interact with one another.
2. Periodicity: Any visit to state *x* with period *d* occurs *k* number of times.
  (18) $$\begin{array}{c} k=gcd\{n>0:pr\left(Y_{n}=x\;|\;Y_{0}=x\right)>0\}, \end{array}$$
  where gcd is the greatest common divisor. Assuming it is easy to visit a state at *k* time step, the state is aperiodic if k=1. Contrarily, the state is periodic if k>1.
3. Transient: A state is transient if we start in state *x* and there is a nonzero probability that we return to *x*.
4. Recurrence: If the number of visit to a state is infinite, then recurrence state has occurred.
5. Absorbing: If one stays in a state, then it is impossible to leave the state; hence, an absorbing state has occurred. Thus, a state *x* is absorbing if $pr_{x,x}=1$ and $pr_{x,y}=0$, x≠y.

## 5. Simulations and Discussion

**Figure 6:**
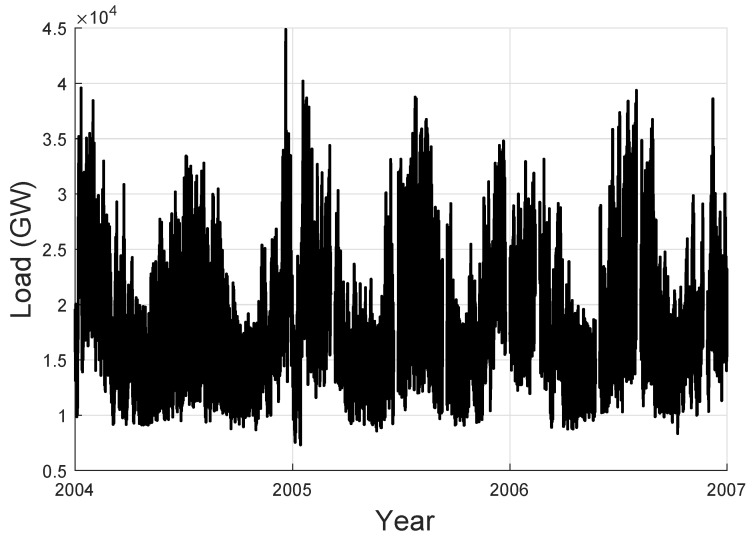
Data from GEFCom2012 load data.

The performance of our proposed AR-MTLF was tested using a dataset comprising 5000 residential customers taken from GEFCom2012 [23]. The dataset is the hourly four-year electrical loads and temperature data across 21 zones of the US utility. It is split into training set (2004–2005), validation set (2006), and testing set (2007). For example, Figure 6 shows the electricity load dataset for Zone 1 ($Z_{1}$). Note that the historical datasets used for the simulations were chosen because they are widely used in the forecasting community [46,47]. All simulations were performed in MATLAB 2018 using a personal computer with 64-bit processor and 8 GB RAM.

To show the performance of AR-MTLF model, we compared its forecasting results with AFC-STLF-, ANN-, NB-, KNN-, SVR-, and ensemble-based forecasting models. For interested readers, the details of these algorithms are discussed in the work by [48]. We discuss the simulation results in the following subsections. Section 5.1 presents the discussions of hourly load forecasting. Section 5.2 provides discussions of seasonal load forecasting. Section 5.3 presents the simulation results based on performance evaluations in terms of forecasting error and convergence rate. Section 5.5 provides the results of consumers’ load consumption dynamics. We considered Zones 1–6 ($Z_{1}$–$Z_{6}$) for further evaluations.

As shown in Figure 3, the total number of elements in the MI-based FS is 2500 using Equation (6). Each value of discrete random variables is derived using Equations (2) and (4). We obtain the time lag data from the temperature data and moving average using Equation (1). We define a threshold of 0.05 for the proposed MI-based FS method. Note that the threshold value of 0.05 is derived from the median of the normalized dataset. Candidate sets greater than the defined threshold are selected as the irrelevant features; otherwise, they are selected as the redundant features. The selected features are used by the forecasting model and the entire process is described in Figure 2. Our proposed MI-based FS method is used to solve the probability distribution of the joint entropy variables and discretized continuous features into 15 partitions. Table 4 shows the probability results for MI of the 15 binary partitions. In the table, it is observed that binary partitions 1–4 and 9–12 have probabilities of zeros, which means that the feature variables are independent. On the other hand, partitions 5–8 and 13–15 have probabilities of 0.91, which means that the feature variables are slightly related. In Table 5, we denote $F_{1}$ as the historical data, while $F_{2}$, $F_{3}$, and $F_{4}$ are denoted as the target value, mean, and moving average value, respectively. For example, when features $F_{1}$ and $F_{2}$ are selected, we evaluate $F_{3}$ and $F_{4}$ by making an equivalent combination of $C=\{F_{1},F_{2},F_{3},F_{4}\}$. Because the combinations of $F_{1}$, $F_{2}$, $F_{3}$, and $F_{4}$ are (0,0,0,0),(0,0,0,1),(0,0,1,0),(0,0,1,1),(0,1,0,0),(0,1,0,1),(0,1,1,0),(0,1,1,1),(1,0,0,1),(1,0,0,0),(1,0,1,0),(1,0,1,1),(1,1,0,0),(1,1,0,1),(1,1,1,0),(1,1,1,1), we assign 1=(0,0,0,0), 2=(0,0,0,1), 3=(0,0,1,0), 4=(0,0,1,1), 5=(0,1,0,0) and so on until 15=(1,1,1,1). If $F_{3}>F_{4}$, then $F_{3}$ will be selected when binary is 1; however, if $MI(C,F_{3})=0.0$ and $MI(C,F_{4})=0.91$, it implies that $F_{4}$ is more relevant to *C* than $F_{3}$. Nevertheless, $F_{4}$ is redundant to the combination of *C*, while $F_{3}$ and *C* are complement to each other (i.e, $MI\left(C,F_{3}\right)=F_{3}=0.0$). Note that this approach is not restricted to the selection of a single feature; multiple features can be selected as well. In addition, the performance of the proposed MI-based FS method depends on the forecasting model.

### 5.1. Hourly Load Forecasting

**Figure 7:**
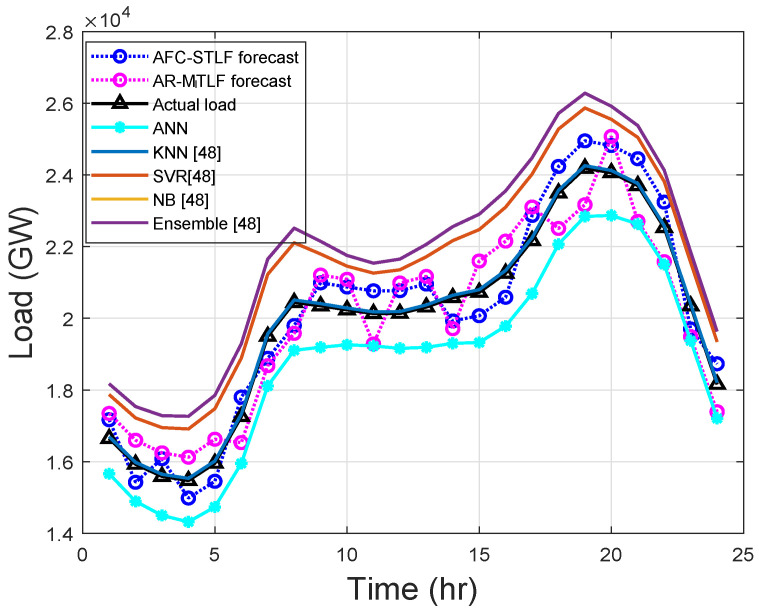
Hourly load forecasting.

The hourly demand for electricity has periodic trends that can be hourly, daily, weekly, monthly, and yearly, which immediately creates the calendar variables. Figure 7 shows the 24-h load forecasting of $Z_{1}$. From the results, it is observed that SVR and ensemble models over forecast the actual load; the reasons are explained in Section 5.2.

KNN and NB models do not learn the actual load, but only memorize it. AR-MTLF and AFC-STLF forecasts are not far from the actual load. It is observed that there are slight rises and falls in forecasted values with the actual load, which is due to consumers’ behaviors. The results show that, at the start of the day, consumer’s electrical consumption begins to rise and fall, alternatively, until the peak hours and suddenly drops after peak hours. Thus, it illustrates the behavior of electrical energy consumption for a consumer.

### 5.2. Load Forecasting Based on Seasons

**Figure 8:**
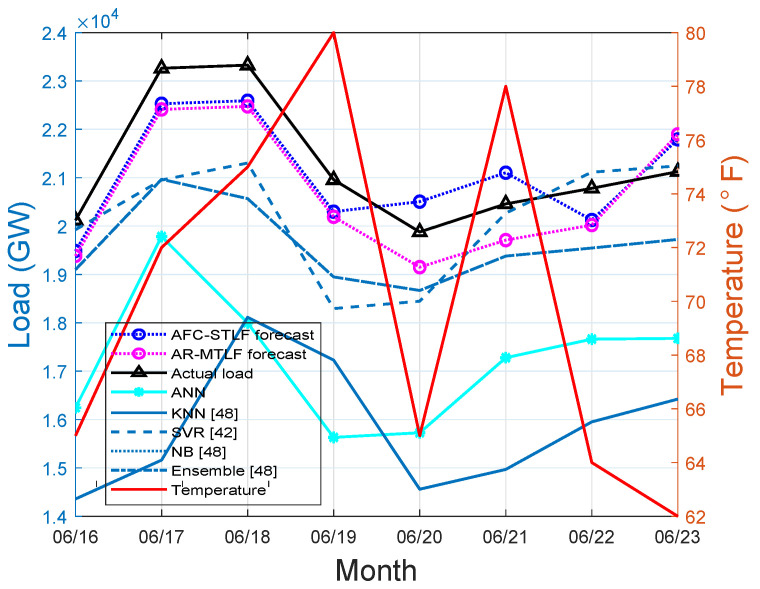
Summer load forecasting.

Figure 8 reports that the actual load and forecasted values of our proposed model have similar pattern for a summer week. The usage of electrical load consumption observed during the weekend (16–18 June 2007) is higher than the electrical load consumption during the working days, which occur when cooling loads are mostly active. The sudden decrease of consumers’ load means that the utility restricts the use of power by means of switching off the energy supply in these periods (20–23 June). Another plausible explanation could be that a higher temperature can spur load demand as the air conditioning continues to operate.

**Figure 9:**
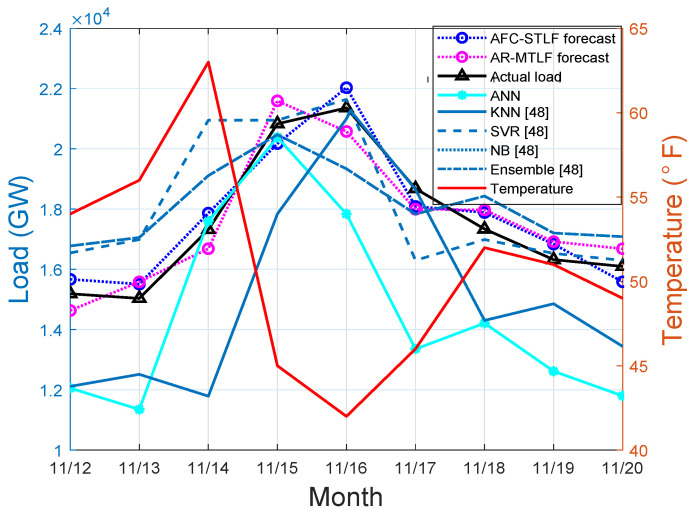
Winter load forecasting.

On the other hand, results in Figure 9 show that heat pumps or other heating devices continue to operate during 15–17 November of the severe winter periods. The continuous use of heating equipment increases the electrical energy consumption, which is observed during the periods of 15–20 November. From the figure, low electrical energy consumption is observed during the start of the week, where the temperature is high, thus, people did not use the heating equipment.

**Figure 10:**
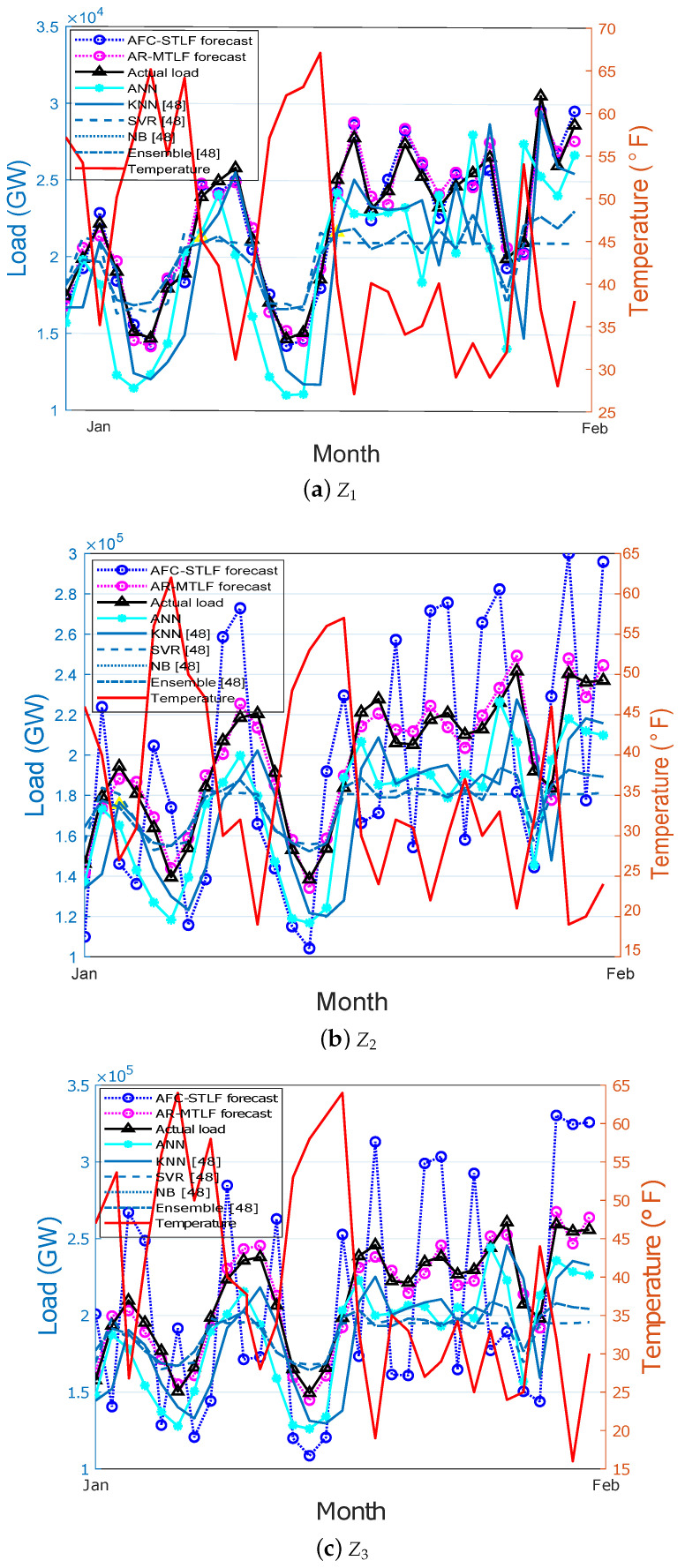

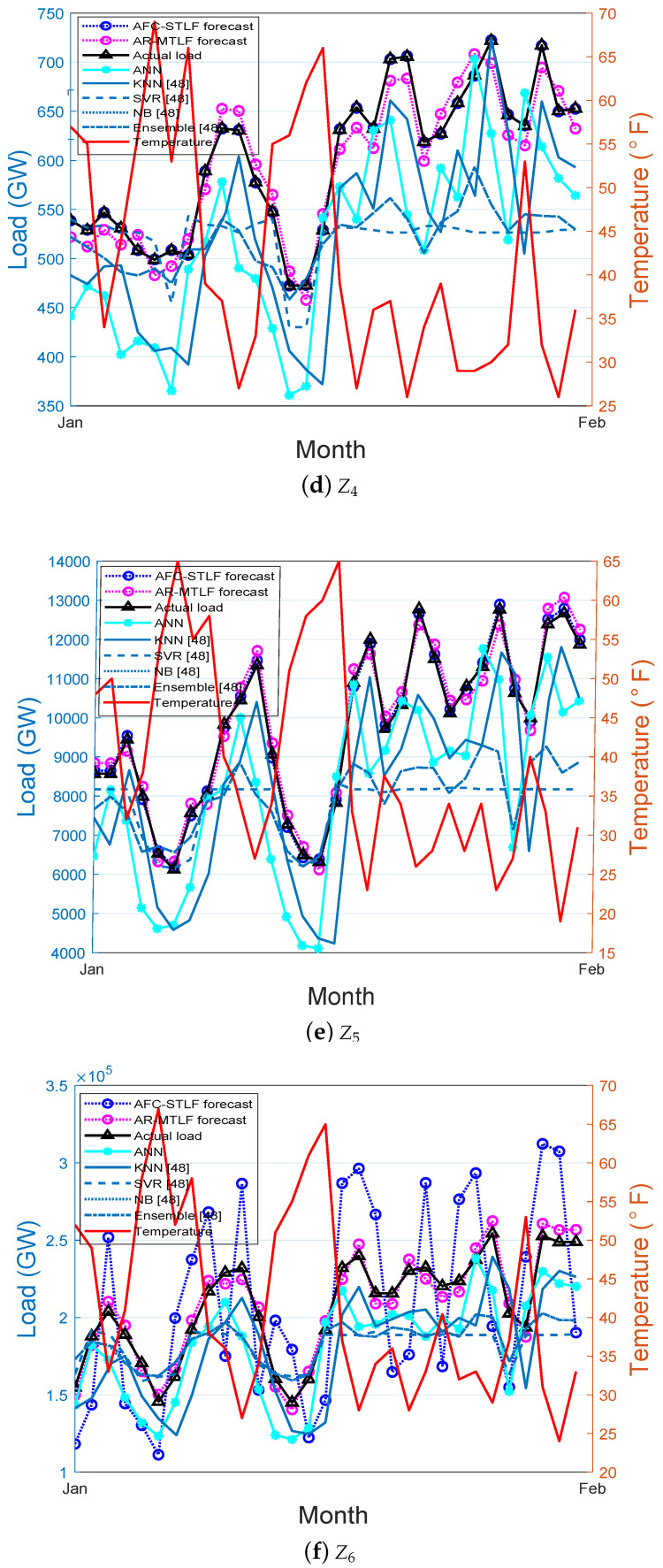
Monthly load forecasting by different zones (**a**–**f**) $Z_{1}$–$Z_{6}$.

Figure 10a–f show the monthly electricity load forecasting for the six zones. In the figure, different electricity load and temperature patterns are observed for the zones. At the end of the month of February, the electrical energy consumption is high for all zones. This is due to the frequent use of high electrical energy consuming equipment. From the results, it is observed that our proposed AR-MTLF model has more similarity with the actual load pattern as compared to the forecasted load values of other models.

**Figure 11:**
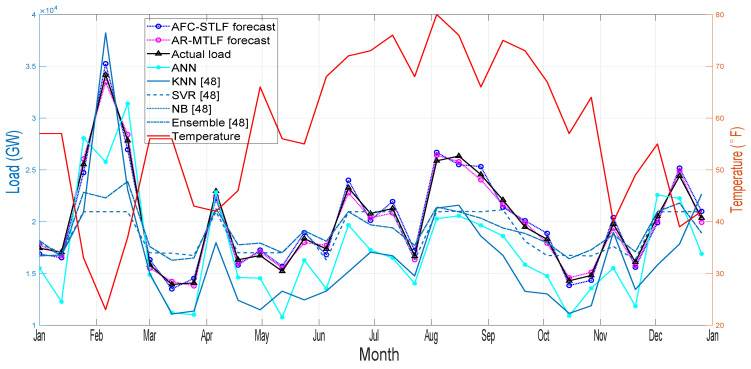
Month ahead load forecasting for one year.

Figure 11 shows the month ahead hourly load forecasting for a year. From the results, it is observed that high electrical energy consumption during the extreme winter and summer periods are influenced by the rise and fall of temperatures. The results of the forecasting clearly show that our proposed model AR-MTLF outperforms the other models. Note that the NB model could not perform well due to the following reasons. NB model depends on labels that assume the shape of the distribution, which means that two features are independent of the output class. In addition, the NB model has continuous features; trying to make them discrete, a lot of information is lost. Furthermore, in the NB model, the classes may be unbalanced, since the method of deriving class labels is based on assumption.

In the ANN model, curse of dimensionality problem is observed, where the approximation result is independent of the dimensions of the input space. Other problems of ANN model may be low convergence, less generalization, and over-fitting. The KNN model, by our assumption, suffers learning issue. In addition, it uses training data for its classification and the wrong choice of selecting the value of k in KNN can affect the results. The ensemble model, on the other hand, has a diversity problem in training the dataset of the six zones. In the SVR model, we select the kernel for the model based on trial and error. We noticed that, as the sample sizes increases, SVR becomes inefficient. The simulation results confirm that our proposed AR-MTLF achieves higher accuracy than the AFC-STLF using the proposed modified MI FS technique as well as deep learning method. This further indicates the importance of our proposed model to resolve the MTLF problem with respect to forecasting accuracy and convergence rate. In addition, the benefits of our scheme over the existing schemes is that it does not memorize the training dataset and can also be implemented on devices with low-memory. Furthermore, our proposed scheme is scalable for large datasets unlike ANN and SVR methods. The multi-layer state of our proposed scheme does not suffer from limited representational power of ANN and ensemble methods. Besides, once data are trained, our proposed scheme allows additional layers to be added.

### 5.3. Proposed Model’s Performance Evaluations

**Figure 12:**
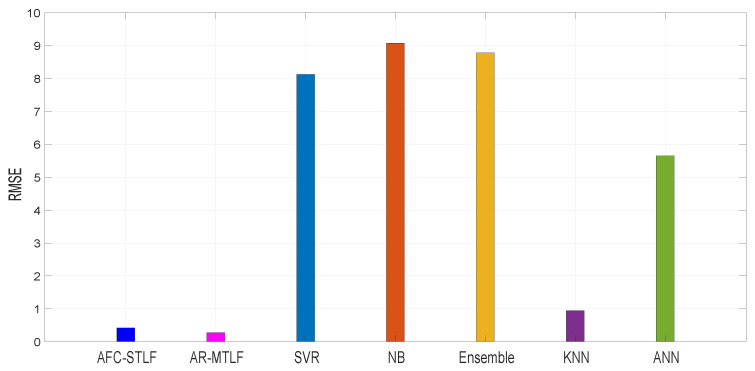
Comparison of forecasting accuracy with existing models for test data of 2007 ($Z_{1}$).

RMSE is used to measure the accuracy of the forecasting in a model; a smaller RMSE means higher accuracy of forecasting model. Figure 12 shows the forecasting accuracy of existing models: RMSE = 8.22 for SVR, RMSE = 9.01 for NB, RMSE = 8.92 for Ensemble, RMSE = 0.98 for KNN, and RMSE = 5.81 for ANN. It is noticed that the AFC-STLF model has RMSE = 0.42, higher than RMSE = 0.32 for our proposed AR-MTLF model. The results show that our proposed model outperforms the existing models with the smallest RMSE value.

Table 6 shows the RMSE values for the six zones. From the results, RMSE values show that our proposed AR-MTLF model is by far the most accurate model as compared to other existing models. However, in $Z_{4}$, AFC-STLF model is by far the most accurate model for that particular zone as compared to the other existing models.

**Figure 13:**
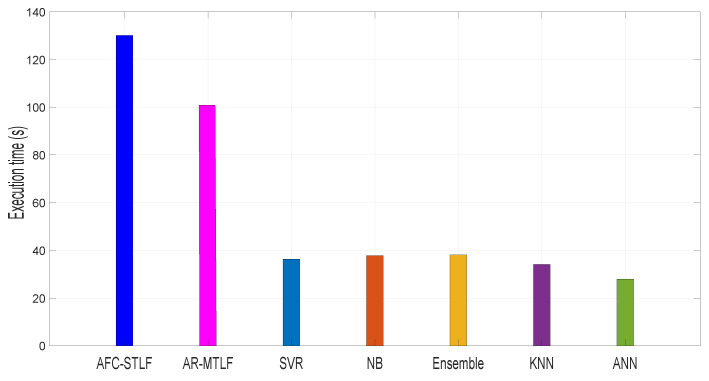
Comparison of execution time with existing models ($Z_{1}$).

Figure 13 depicts the comparison of the proposed model with the existing models based on execution time. The execution time observed for our proposed AR-MTLF and AFC-STLF models are 100.00 and 125.50 s, respectively. AR-MTLF model minimizes execution time because the optimizer finds the global optimal solution with less execution time as compared to AFC-STLF model. However, there is no general way to detect that a solution has achieved a global solution. Moreover, in some cases, the fitness of global optimum or some bounds on it value may be known. In our case, we consider the optimality of the solution by inspecting the fitness value. In addition, we consider the number of iterations and the stopping criteria of the Jaya-based optimization algorithm to determine if the optimum solution is reached. Furthermore, if the current solutions are better than the formal solutions, the current solutions are considered as the optimum solutions after the stopping criteria are reached. In the figure, other existing models have less execution time as compared to the AR-MTLF and AFC-STLF models that incorporate optimization techniques such as Jaya and mEDE.

Table 7 shows the execution time in seconds for all models. From the results, the models with highest execution time are observed from AR-MTLF and AFC-STLF. The high execution time occurs because of the extra execution time in implementing FS and optimization algorithms. Note that there is trade off between forecasting accuracy and execution time, as shown in Table 6 and Table 7, respectively.

**Figure 14:**
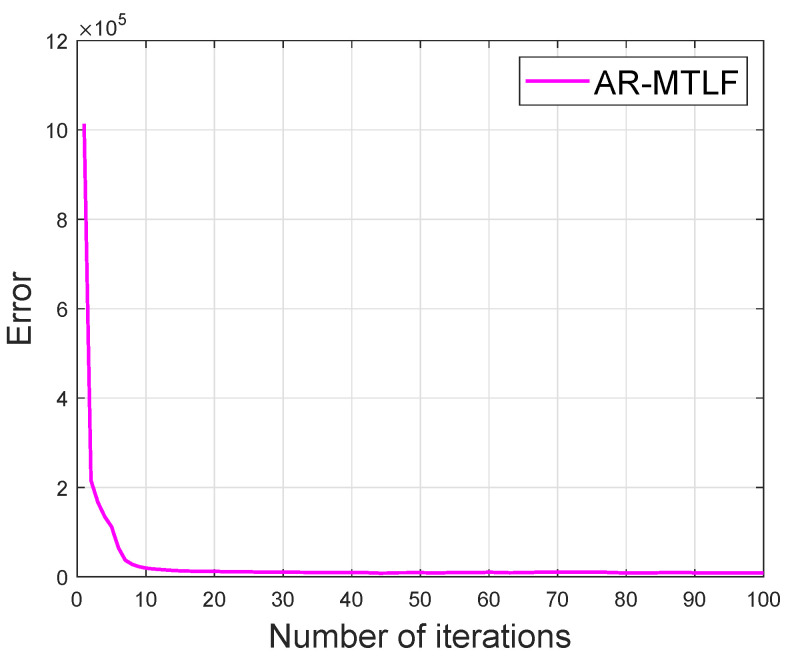
Error versus number of iterations.

Figure 14 shows that the convergence of our proposed AR-MTLF model. The convergence starts at 20th iteration. The results clearly indicate that the model achieves a global optimum solution within a reasonable number of iterations. In addition, our proposed AR-MTLF FS process reflects good performance for the lagged temperature data and average observed data.

**Figure 15:**
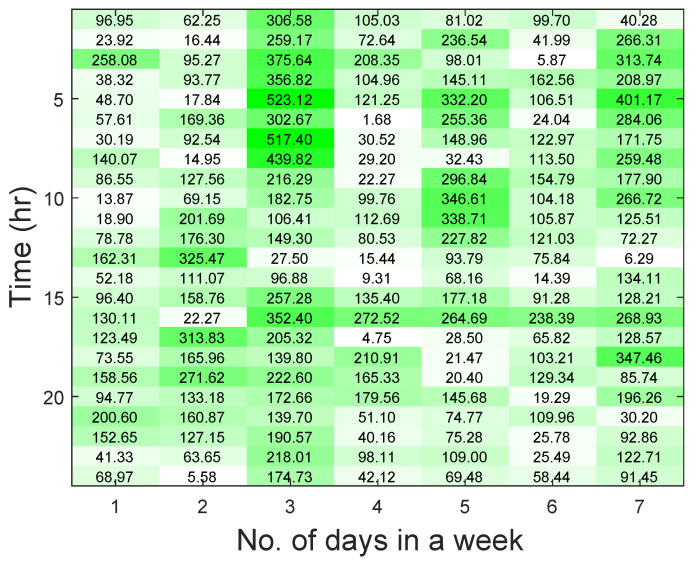
Heat map reporting the RMSE values. White color indicates RMSE range of 0–40; light green color indicates RMSE range of 41–200; dark green color indicates RMSE of 201–500.

We show the RMSE values for the different time periods using a heat map in Figure 15. The heat map shows the first one week to avoid verbosity. The lowest RMSE value indicates that the forecasting result is accurate in that period. For example, in the heat map, if h = 17 and d = 4, RMSE value is 4.75.

### 5.4. Operational Forecast

**Figure 16:**
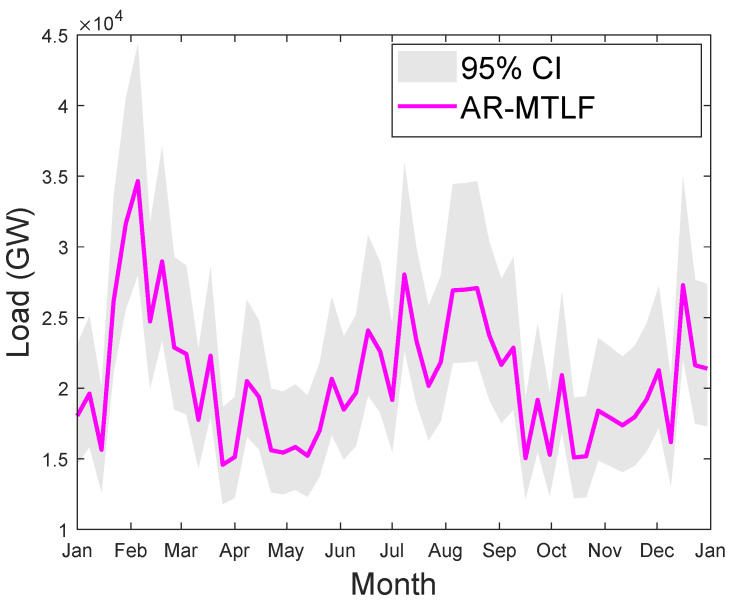
Seven days of consecutive MTLF distribution of load from January to December 2007 using the actual temperature value. The gray plot indicates the confident interval (CI) of 95%.

**Figure 17:**
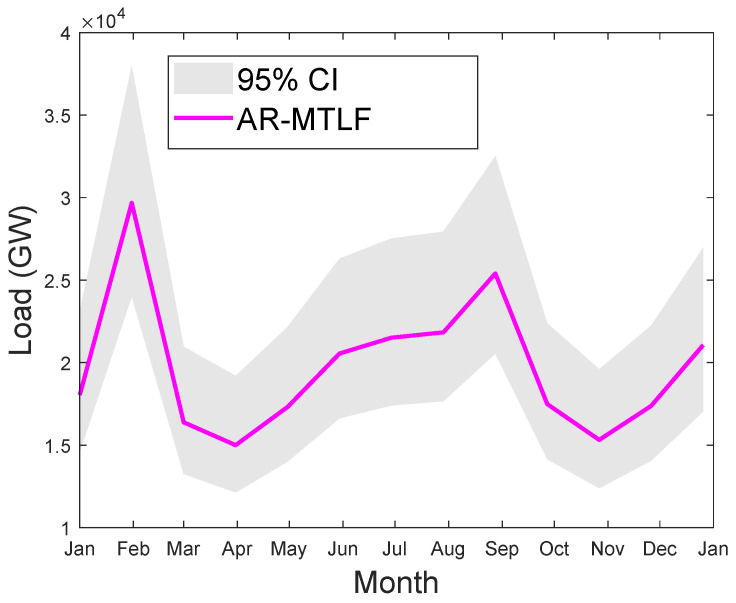
Thirty days of consecutive MTLF distribution of load from January to December 2007 using the average distribution of electrical load and temperature values. The gray plot indicates the confident interval (CI) of 95%.

We consider the MTLF distribution of consumers’ electricity load for seven consecutive days using the temperature values for a year. However, the forecasting results do not reflect the uncertainty in the temperature; hence, the confident interval (CI) is smaller. CI enables us to know the range of values for the given distribution. Since we are concerned with capturing the true distribution of the consumers’ electrical load consumption, a wider interval would be much better. Thus, the accuracy of forecasting will increase with the increase in CI; however, precision may decrease. Figure 16 shows the MTLF distribution of consumers’ electrical load for seven consecutive days. It is observed that our proposed AR-MTLF model ensures accuracy and precision. We consider also the true distribution of the consumers’ electrical load, while the certainty of the future temperature is unknown. To resolve this issue, we make an average between the consumers’ electrical load and the temperature to derive a new MTLF distribution [28]. Note that we perform forecasting on new MTLF distribution and examine the distribution of 30 consecutive days with CI of 95%, as shown Figure 17. It is observed that CI is much larger than CI of Figure 16, which means that the true values of temperature are known.

### 5.5. Consumers Consumption Dynamic

A way of determining the unique behavior of consumers’ consumption is to partition them into groups. An adaptive k-means is applied to the entire dataset and, afterwards, the number of k-centroid is obtained. Five groups are formed from k-centroid, which are distinguished in the bar chart shown in Figure 5. Each bar in the figure represents electricity consumption. Consumers with the same behavior are placed in the same bar. It can be observed that the energy consumption of different bars is not equally distributed. By our assumption, 90% of the consumption belongs to the last large bar, whereas the rest is distributed in the other four bars. In addition, consumers’ consumption in the same bar relates to their electricity consumption behaviors over a specific period of time.

**Figure 18:**
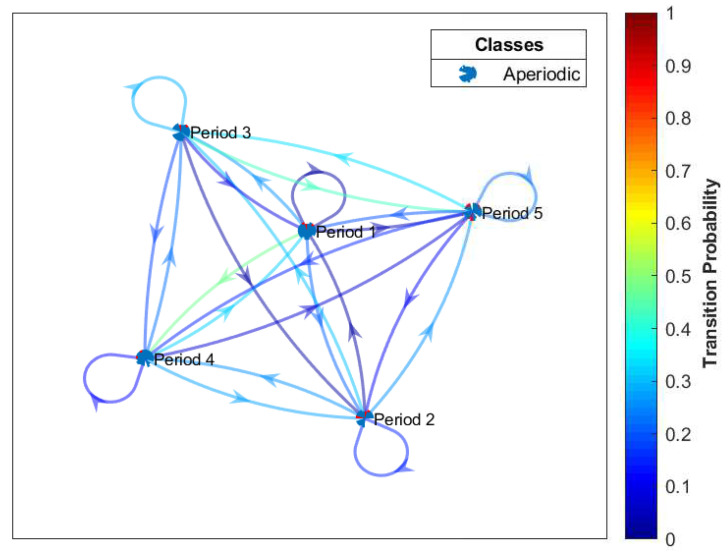
Probability of transition using edge colors.

We are rarely concerned with the consumers’ load consumption dynamic characteristics in all periods; we rather concentrate on a specific period of time. A DTMC process is employed on the sequence of demand response of dynamic consumers’ load consumption behavior from one state to another. This is possible only if we consider the grouping of consumers’ consumption in different adjacent periods. Figure 18 shows the transition states of the consumers’ load consumption behaviors in different adjacent periods. We observe that the consumers’ load consumption behaviors are viewed in five adjacent periods: let Period 1 be the lowest consumption; Period 2 be the low consumption; Period 3 be the average consumption; Period 4 be the high consumption; and Period 5 be the extremely high consumption. It is worth-mentioning that all periods belong to the aperiodic class. All periods are transient and the dynamic behaviors of consumers show much diversity since consumers’ load consumption depend on temperature as well as the activities of daily living (ADL), i.e., classifying activities and tasks of consumers in the house. The consumer can move from one state to another without being absorbed in that state.

**Figure 19:**
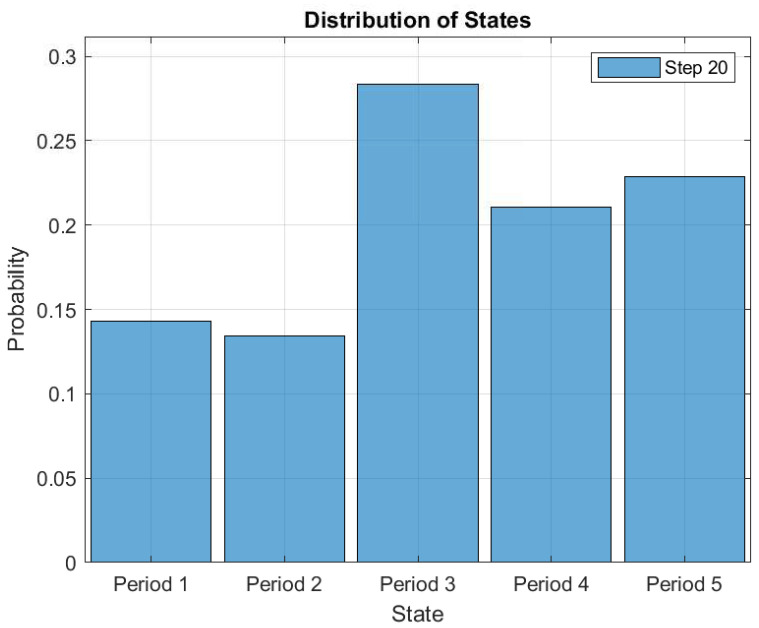
Distribution probability of each state after 20 simulation iterations.

This makes it possible to study consumers’ load consumption behavioral patterns. Figure 19 shows the probability distributions of each state after 20 simulation iterations. In the figure, it is observed that the consumers’ average energy consumption probability of 0.27 is the highest as compared to the other classes of energy consumption. It means that the consumers have applied the demand response strategy to reduce their cost of electricity. Because of the different events of a day, consumers may change their states from average consumption to extremely high consumption to satisfy their load demands. To avoid being charged at a significantly higher rate than normal for all energy consumption, consumers immediately change their states from extremely high consumption to high consumption and subsequently to very low consumption. The ability to measure the electricity consumption of consumers throughout the period can provide an insight of the consumers’ ADL. This helps both the consumer and utility in proper electricity management and planning.

## 6. Conclusions

MTLF is an emerging paradigm for electricity load forecasting. Many methods of MTLF exist in the literature that focus on the forecasting of daily peak load, daily energy consumption, and annual peak load consumption. However, this work focuses on month ahead hourly electricity load forecasting, which is important for grid’s maintenance planning and harmonizing energy sharing arrangement. In addition, a modified MI-based FS model is proposed to eliminate redundancy and irrelevancy of features from the dataset. The proposed AR-MTLF model is used for electricity load forecasting, which resolves the limitations of AFC-STLF model through its ability to learn from massive amounts of data with less computational overhead. From the forecasting results, the relationship between temperature and electricity loads is examined. The existing model AFC-STLF focuses on day-to-day industrial smart grid operations using the lagged input samples. However, month ahead hourly load forecasting is not considered in the AFC-STLF model. In FS process of AFC-STLF, the downsized inputs do not reduce the training time; here, information loss is observed. This is due to the unstable convergence of the mEDE and inefficiency of the model in learning from massive amount of data. AR-MTLF model is proposed to overcome the stated trade offs. The newly proposed AR-MTLF achieves approximately 99.68% accuracy as compared to AFC-STLF with 99.58% accuracy. In addition, AR-MTLF model achieves execution time reduction up to 54.64% as compared to 46.12% of AFC-STLF. Furthermore, we compared our proposed model with KNN, ANN, NB, SVR, and Ensemble forecasting models, and the results clearly report that our model outperforms its counterparts.

This paper also proposes a novel approach to group consumers based on their energy consumption behaviors. Since the behaviors of consumers change from time to time, the need to examine their behaviors has become imperative to ensure proper management and planning. A DTMC is performed to uncover the typical electricity consumption dynamics and divide consumers into several distinct groups using the adaptive k-means.

As consumers do not follow well defined energy consumption patterns, there is a tendency that consumers’ behavior will be repeated. Thus, if we can learn consumers’ behavior, we may be able to deduce their next behavior. Based on this fact, our future work aims to employ reinforcement learning for real-time feedback. With this approach, consumers can be seen as the set of actions established over time.

## Figures and Tables

**Table 1:** The summary of related work.

S/N	Size of Dataset (year)	Time Resolution (h)	FS	Techniques	Objective(s)	References
1.	4.5	1	✓	SVR	STLF	[8]
2.	3	2	✓	FPR	STLF	[9]
3.	1	3 min	✓	GABICS-DFS-ELM	STLF	[10]
4.	2	1	✓	AIS	STLF	[11]
5.	1	1	✓	WLSSVM-SWA	STLF	[12]
6.	1	30 min	✓	PRNN	STLF	[13]
7.	1	1	✓	IENN	STLF	[14]
8.	2	30 min	✓	LSTM-RNN	STLF	[16]
9.	2	5 min	✓	NN, LR and MTR	VTLF	[17]
10.	4	24	✓	MRMRMS	STLF and STPF	[24]
11.	4	15 min	-	CRBM and FCRBM	STLF	[29]
12.	1.5	30 min	-	k-means	LTLF	[30]
13.	4	6	-	Clustering technique and Bayesian network	STLF + LTLF	[31]
14.	1	30 min	-	OS-ELM	STLF	[19]
15.	2	15 min	✓	Inter cluster technique	VSTLF	[32]
16.	1	1	-	Cluster analysis, association analysis and decision tree	STLF	[33]
17.	4.5	1	✓	CRBM, adaptive k-means and DTMC	MTLF	Proposed scheme

✓, considered; -, not considered.

**Table 2:** Simulation parameters.

Parameter	Value
Population size (Jaya)	24
Number of decision variables (Jaya)	2
Maximum iterations	100
Maximum bound (Jaya)	0.9
Minimum bound (Jaya)	0.1
Number hidden layers (CRBM)	10
Learning rate (CRBM)	0.001
Weight decay (CRBM)	0.0002
Momentum (CRBM)	0.5

**Table 3:** Consumer electricity consumption behavior transition matrix.

x/i	*1*	*2*	*3*	*4*	*5*
***1***	0.1458	0.1458	0.2500	0.0833	0.3750
***2***	0.0517	0.2931	0.0862	0.3793	0.1897
***3***	0.1647	0.2824	0.1059	0.2824	0.1647
***4***	0.0877	0.0877	0.4561	0.2895	0.0789
***5***	0.2203	0.0000	0.1356	0.5254	0.1186

**Table 4:** MI for the four joint discrete random variables.

1	2	3	4	5	6	7	8	9	10	11	12	13	14	15	16
0.0	0.0	0.0	0.0	0.91	0.91	0.91	0.91	0.0	0.0	0.0	0.0	0.91	0.91	0.91	0.91

**Table 5:** Joint probability of the individual value of FS. $F_{1}$ denotes the historical data, while $F_{2}$, $F_{3}$, and $F_{4}$ denote the target value, mean, and moving average value, respectively.

Binary	$F_{1}$	$F_{2}$	$F_{3}$	$F_{4}$
0	0.6667	0.0000	0.4986	0.5002
1	0.6667	1.0000	0.5014	0.4998

**Table 6:** Comparisons of forecasting accuracies for the selected zones ($Z_{1}$–$Z_{6}$).

*Z*	AR-MTLF	AFC-STLF	SVR	NB	Ensemble	KNN	ANN
1	0.32	0.42	8.22	9.01	8.92	0.98	5.81
2	0.35	11.40	1.03	5.52	5.68	11.87	10.12
3	0.40	13.30	1.52	5.88	5.92	13.02	11.54
4	0.31	0.25	0.58	0.48	2.45	0.30	3.18
5	0.40	0.58	1.10	0.83	0.78	0.72	1.53
6	0.51	11.81	1.30	7.02	6.37	11.0	9.92

**Table 7:** Comparisons of execution time in seconds for the selected zones ($Z_{1}$–$Z_{6}$).

*Z*	AR-MTLF	AFC-STLF	SVR	NB	Ensemble	KNN	ANN
1	100.00	125.50	37.52	39.20	38.10	38.35	30.21
2	142.01	146.76	36.21	39.27	39.32	37.22	35.90
3	161.32	172.34	35.89	38.23	38.322	35.22	30.21
4	158.23	170.39	42.91	43.82	42.49	40.21	35.32
5	178.39	184.87	38.21	39.88	40.44	37.92	30.77
6	150.45	160.76	39.87	40.32	40.88	38.22	30.55

## Abbreviations

The following abbreviations are used in this paper:

ANN	Artificial neural network
AFC-STLF	Accurate fast converging short-term load forecasting
AG	Antigen
AIS	Artificial immune system
AR-MTLF	An accurate and robust medium-term load forecasting
CRBM	Condition restricted Boltzmann machine
DFS	Date-framework strategy
DTMC	Discrete time Markov chain
DWT-IR	Discrete wavelet transform and inconsistency rate
EMD	Empirical mode decomposition
ELM	Extreme learning machine
FCRMB	Factor CRBM
FPR	Fuzzy polynomial regression
FS	Feature selection
GA	Genetic algorithm
GABICS	GA binary improved cuckoo search
IENN	Improved Elman neural network
KNN	K-nearest neighbors
LR	Linear regression
LSSVM	Least square support vector machine
LSTM	Long short term memory
LTLF	Long-term load forecasting
mEDE	Modified enhanced differential evolution algorithm
MI	Mutual information
MRMRPC	Maximizes the relevancy and minimizes the redundancy on Pearson’s correlation
MRMRMS	Maximizes relevancy and minimizes redundancy and maximizes synergy of candidate features
MTLF	Medium-term load forecasting
MTR	Model tree rules
NB	Naive Bayes
OS-ELM	online sequence ELM
PRNN	Pyramid system and recurrent neural networks
RMSE	Root mean square error
SWA	Sperm whale algorithm
SVR	Support vector regression
STLF	Short-term load forecasting
STPF	Short-term price forecasting
SWEMD	Sliding window EMD
VSTLF	Very short-term load forecasting
WLSSVM	Wavelet least square support vector machine
